# The Scandinavian journal of trauma, resuscitation and emergency medicine – grown up at last

**DOI:** 10.1186/1757-7241-16-1

**Published:** 2008-07-21

**Authors:** Hans Morten Lossius

**Affiliations:** 1 Director of Research, Norwegian Air Ambulance Foundation, Norway

## 

*The Scandinavian Journal of Trauma, Resuscitation, and Emergency Medicine (SJTREM) *was launched in 1994 as *Akuttjournalen*. From being a national, humble magazine directed to anaesthesiologists working in the Norwegian air ambulance service, the journal has developed to being a peer-reviewed, international journal directed at all health professionals involved in pre- and in-hospital emergency medicine, critical care and trauma management.

Over the years, *SJTREM *has gained valuable experience through both funding several international conferences (i.e. TraumaCare 2002, HLR 2003, The Scandinavian Update on Trauma, Resuscitation, and Emergency Medicine 2005 and 2007 – to mention but a few), and publishing widely on topics in subspecialties covered by its scope [1].

Today, *SJTREM *is the official journal of The Scandinavian Networking Group on Trauma and Emergency Management (SCANTEM) [2], and 9 more societies involved in trauma, resuscitation, and emergency medicine in Scandinavia. Although primarily directed at the Scandinavian audience, the articles published reflect the journal's considerable international orientation. Our large and distinguished Editorial Board represents several different countries, including UK, Germany, Australia, and the US.

When you read this, a new and significant step in the history of *SJTREM *is achieved. The journal is now published as an open access online journal in cooperation with BioMed Central. This means that articles will be published online immediately upon acceptance (after peer-review) and soon after listed in PubMed Central, the US National Library of Medicine's full-text repository of life science literature, and hence indexed in PubMed.

*SJTREM *has chosen open access publishing for several reasons. Articles are freely and universally accessible online, thus articles are highly visible and read by a wide audience. The authors hold copyright for their work and grant anyone the right to reproduce and disseminate the article provided that it is correctly cited, in accordance with BioMed Central's open access license agreement [3]. Besides PubMed Central, the journal's articles are archived in repositories at the University of Potsdam in Germany, at INIST in France and in e-Depot, the National Library of the Netherlands' digital archive of all electronic publications.

Thanks to substantial funding from The Norwegian Air Ambulance Foundation and The Laerdal Foundation for Acute Medicine, all article-processing charges are covered by the journal. The results of scientific research, as well as clinical experience and commentaries published with *SJTREM *will be available free of charge to the whole emergency medicine community, both authors and readers.

The clinical ideology of *SJTREM *is based on two concepts; The Formula of Survival [4] and The Chain of Survival [5]. The Formula of Survival illustrates how the interaction between medical science, educational efficiency and local organization does affect outcome. All three factors have equal importance, and deserve equal focus. The Chain of Survival emphasizes how all elements and personnel involved, from time of injury or illness and throughout the course of the treatment, influence the outcome. Cooperation, communication and collaboration across educational and professional borders are a prerequisite to success.

The scientific ideology of *SJTREM *emphasizes innovation. Although randomized controlled trials (RCT) are accepted as a gold standard, the method has significant limitations in emergency medical research. The interpretation of RCTs conducted in a multi-factorial, low incidence, "real time" emergency medical world requires caution. The characteristics and number of the study population, setting, connection between cause and effect, and the true implications of the study must be carefully considered. *SJTREM *believe that the best results in solving problems will be achieved by looking at them from different angles, using various approaches. In this, qualitative and quantitative methods complement each other. We encourage inventiveness and willingness to explore untraditional research methodology.

*SJTREM *has a large and well qualified, international referee board. We operate a closed peer-review system and aim to reach a first decision to accept or reject a manuscript within six weeks of submission.

Pre-hospital and immediate in-hospital emergency care involves a broad spectrum of disciplines, specialties and skills, which may differ significantly in structure, resources and operation between different systems and nations. For years, an unsolved issue has been when advanced life support (ALS) should be seen as preferable to basic life support (BLS), especially in trauma patients [6,7]. The line is often drawn on the steps of the emergency department, or in some cases the ALS is withheld until the patients pass the door of super-specialised medical services deep inside the hospital building. *SJTREM *believe that to clarify this complex issue we require research aiming to separate the impact of organisational structure from patients' patho-physiology and need for emergency medical interventions. Further, the isolated effect of provider competence, and the most effective ways to gain and retain competence must be deeply explored.

The *Scandinavian Journal of Trauma, Resuscitation, and Emergency Medicine *look forward to linking science and everyday work helping us all to build the pre- and in-hospital emergency medical systems for the future.

## Competing interests

The author declares that they have no competing interests.
